# Cryptic Diversity in the Ubiquist Species *Parisotoma notabilis* (Collembola, Isotomidae): A Long-Used Chimeric Species?

**DOI:** 10.1371/journal.pone.0046056

**Published:** 2012-09-26

**Authors:** David Porco, Mikhail Potapov, Anne Bedos, Galina Busmachiu, Wanda M. Weiner, Salah Hamra-Kroua, Louis Deharveng

**Affiliations:** 1 Laboratoire d’Ecologie, EA 1293 ECODIV, FED SCALE, Université de Rouen, Mont Saint Aignan, France; 2 Department of Zoology and Ecology, Moscow State Pedagogical University, Moscow, Russia; 3 Museum National d’Histoire Naturelle, UMR7205 ”Origine, Structure et Evolution de la Biodiversité", Paris, France; 4 Institute of Zoology, Academy of Sciences of Moldova, Chisinau, Republic of Moldova; 5 Institute of Systematics and Evolutions of Animals, Polish Academy of Sciences, Kraków, Poland; 6 Laboratoire de Biosystématique et Ecologie des Arthropodes, Université Mentouri Constantine, Faculté SNV, Département de Biologie Animale, Constantine, Algérie; Biodiversity Insitute of Ontario - University of Guelph, Canada

## Abstract

*Parisotoma notabilis* is the most common species of Collembola in Europe and is currently designated as ubiquist. This species has been extensively used in numerous studies and is considered as well characterized on a morphological ground. Despite the homogeneity of its morphology, the sequencing of the barcoding fragment (5′ end of COI) for several populations throughout Europe and North America revealed four distinct genetic lineages. The divergence found between these lineages was similar to the genetic distance among other species of the genus *Parisotoma* included in the analysis. All four lineages have been confirmed by the nuclear gene 28S. This congruence between mitochondrial and nuclear signals, as well as the geographical distribution pattern of lineages observed in Europe, supports the potential specific status of these lineages. Based on specimens from the type locality (Hamburg), the species name was successfully assigned to one of these lineages. This finding raises several problems as *Parisotoma notabilis* has been widely used in many ecological studies. Accumulation of new data for the different lineages detected, especially ecological information and life history traits, is needed to help resolve this situation.

## Introduction

*Parisotoma notabilis* was described by Schäffer in 1896 from Hamburg in Germany. Since then it has been abundantly recorded from various regions in the world [1], [2] and can be considered as the most abundant species of Collembola in the temperate regions of the west Palearctic [2]. Populations reach their highest densities in Northern and Central Europe, decreasing steadily in the Mediterranean, Siberian and Arctic regions. The species is often rare in Mediterranean lowland regions and in high endemism areas such as Slovenia (unpub. observations) or Central Pyrenees [3], [4]. Following the description of several other species of *Parisotoma* [5], [6], [7], the citations from Eastern Palearctic for this species are considered dubious, and the species in its modern definition has not been recorded recently from these localities. The wide distribution of *P. notabilis* can partly be due to its near obligate parthenogenesis [8], [9] which facilitates its rapid installation and spreading in new localities.

*Parisotoma notabilis* is currently considered as well characterized morphologically [10], [11], [12], [7], . It is defined by a combination of characters including 2–5+2–5 eyes, 4+4 postlabial chaetae, an abundant S-chaetotaxy, lower subcoxa of leg I without chaeta, 3 chaetae on each lateral flap of the ventral tube and 2 chaetae on tenaculum. Four synonyms were listed by Potapov [2]: *Isotoma menotabilis* Börner, 1903; *I. delicatula* Brown, 1929; *I. eunotabilis* Folsom 1937; *Desoria monticola* Hao and Huang, 1995. By contrast with its morphological homogeneity, *P. notabilis* is known to be plastic ecologically, and can be found in a broad range of natural or disturbed habitats, along a wide altitudinal range [2]. The species is often favored in disturbed [13], [14], [15] or regenerating habitats [16], [17]. *P. notabilis* has paradoxically been reported to both tolerate and being negatively impacted by toxics such as industrial pollution [18], pesticides [19], [15] and heavy metal [20], [21], [15], [22]. *Parisotoma notabilis* was also considered to be a poor indicator for pH, reacting positively to either low or high pH values [23]. So even if *P. notabilis* is morphologically well defined, the broad range of its ecological and life history traits raises interrogations about its status as a unique specific entity.

In order to explore and test the genetic homogeneity of this ubiquist species, we used the DNA barcoding fragment (5′ COI) which has proved to be informative for congeneric and closely related species delineation in many groups [24], [25], [26] including collembolans [27]. A fragment of a nuclear gene (28S, D2 region) was also sequenced to confirm the COI results.

## Materials and Methods

### Sampling

**Figure 1:**
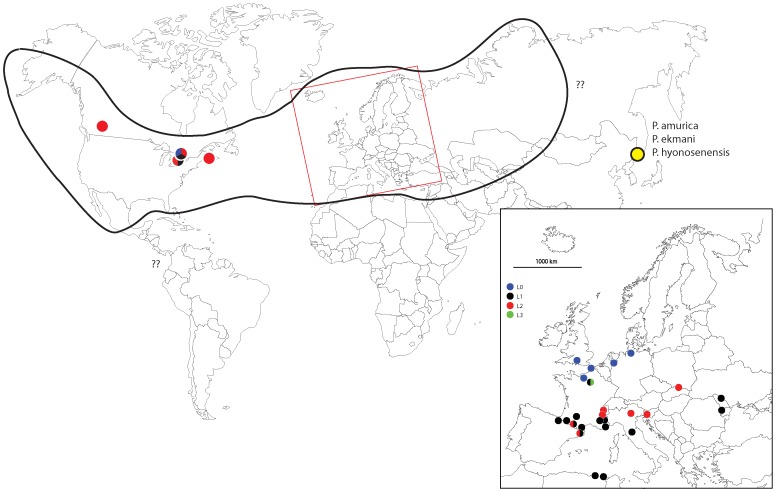
Geographical distribution of *Parisotoma notabilis* lineages analyzed in this study. The global distribution potential limit of the species is showed by a thick line.

252 specimens from 37 populations of *P. notabilis* were sampled both in North America and Europe (Table 1, Fig. 1). The type locality of the species in Hamburg was sampled as well. A largest number of specimens from three localities (Guelph, Paris and Hamburg) were analyzed in order to evaluate the intrapopulational divergence and to test the potential sympatry of lineages. Three other species of *Parisotoma* were added to the dataset to provide a reference for both intraspecific and interspecific genetic variation in other species within the genus: *P. eckmani*, *P. amurica* and *P. hyonosenensis* (Table 1).

**Table 1:** Sampled populations.

Species	Country	Locality	N	Lineages
*Parisotoma notabilis*	Algeria	Edough	1	L1
	Algeria	Collo	1	L1
	Canada	British Columbia, Glacier NP	2	L2
	Canada	New Brunswick, Fundy	1	L2
	Canada	Nova Scotia, Kejimkujik	4	L2
	Canada	Ontario, Grey County	10	L0,L1,L2
	Canada	Ontario, Guelph	25	L1, L2
	Canada	Ontario, Elora	8	L1, L2
	Canada	Ontario, Haliburton	3	L2
	Canada	Ontario, Kawartha	5	L1
	France	Drome, Verclause	4	L1
	France	Seine Maritime	1	L0
	France	Isere, Sarcenas	4	L2
	France	Pas-de-Calais, Wimereux	6	L0
	France	Paris	87	L1, L3
	France	Essonne, Brunoy	2	L1
	France	Pyrenees-Orientales, Corsavy	8	L1
	France	Pyrenees-Orientales, L’Albere	5	L1
	France	Pyrenees-Orientales, Les Cluses	6	L1
	France	Pyrenees-Orientales, Mosset	6	L1
	France	Ariege, Le Port	3	L1, L2
	France	Haute-Garonne, Toulouse	5	L1
	France	Hautes-Pyrenees, Tarbes	5	L1
	France	Alpes-Maritimes, Peille	2	L1
	France	Hautes-Alpes, Aspres-sur-Buech	1	L1
	France	Savoie, Saint-Jean-de-Couz	4	L2
	Germany	Hamburg, Blankenese	17	L0
	Italy	Siena, Vagliali	1	L1
	Italy	Verona, Erbezzo	1	L2
	Moldova	Rezina	2	L1
	Moldova	Baius	2	L1
	Netherlands	Zutphen	3	L0
	Poland	Tatra Mts, Mala Laka valley	1	L2
	Slovenia	Bohinjska Bistrica	2	L2
	Spain	Catalonia, Gerona	6	L1, L2
	Spain	Navarra, Aritzkuren	4	L1
	United Kingdom	England, Hampshire	4	L0
*Parisotoma amurica*	Russia	Primorye Territory, Anisimovka	4	
*Parisotoma ekmani*	Russia	Primorye Territory, Khualaza	3	
*Parisotoma hyonosenensis*	Russia	Primorye Territory, Khualaza	2	

### Molecular Analysis

DNA was extracted from entire specimens in 30 µl of lysis buffer and proteinase K incubated at 56°C overnight. DNA extraction followed a standard automated protocol using 96-well glass fibre plates [28]. Specimens were recovered after DNA extraction using a specially designed work flow allowing further morphological examination [29]. The 5′ region of COI used as a standard DNA barcode was amplified using M13 tailed primers LCO1490 and HCO2198 [30]. Samples that failed to generate an amplicon were subsequently amplified with a pair of internal primers combined with full length ones LepF1-MLepR1 and MLepF1-LepR1 [31]. A standard PCR reaction protocol was used for amplifications, and products were checked on a 2% E-gel 96Agarose (Invitrogen). Unpurified PCR amplicons were sequenced in both directions using M13 tailed primers [32], with products subsequently purified using Agencourt CleanSEQ protocol and processed using BigDye version 3.1 on an ABI 3730 DNA Analyzer (Applied Biosystems). Sequences were assembled with Sequencer 4.5 (GeneCode Corporation, Ann Arbor, MI, USA) and aligned by eye using BIOEDIT version 7.0.5.3 [33].

In addition, for 102 of the sampled *P. notabilis* specimens, a fragment of the D2 region of the nuclear gene 28S of 411 base pairs was amplified and sequenced with the same conditions than COI. The primer pair used was ‘D2coll’ and ‘C2’coll’ designed specifically for Collembola [34]. As we observed no indels in the COI and 28S sequences, sequence alignment was unambiguous. Sequences are publicly available on BOLD in the project ‘DATASET-CRYCOL2’ and on Genbank (GQ373667, GQ373669, GQ373670, GU656217, GU656408–GU656423, HM397729, HM397730, HM397803, HM398181, HM909156, HM909328, HQ559271, HQ559489–HQ559494, HQ942514, HQ942680–HQ942685, HQ943204, HQ943258–HQ943260, HQ943297, HQ943298, JN298119–JN298134, JQ935008–JQ935203, JQ909881–JQ909984).

### Calculations

Distance analyses were performed with MEGA4 software [35]. Neighbor-Joining [36] algorithm with the Kimura-2 parameter model [37] has been used to estimate the genetic distances. The robustness of nodes was evaluated through bootstrap re-analysis of 1000 pseudoreplicates. The trees have been replotted using the online utility iTOL [38].

## Results

**Figure 2:**
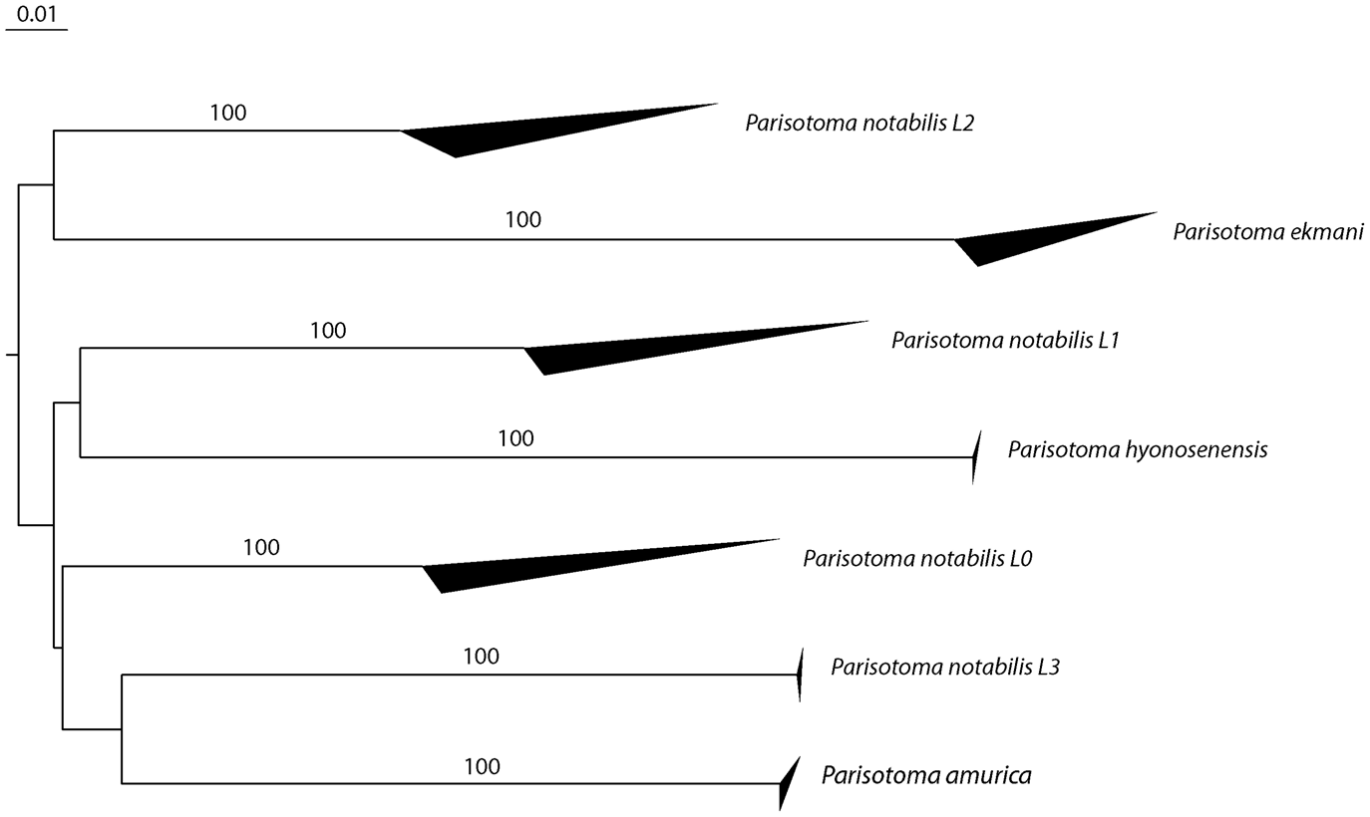
Neighbor-joining K2P distance tree for COI (Bootstrap support values showed on the branches. Upper and lower side of the triangle represent the maximum and minimum of genetic distances within a species).

**Figure 3:**
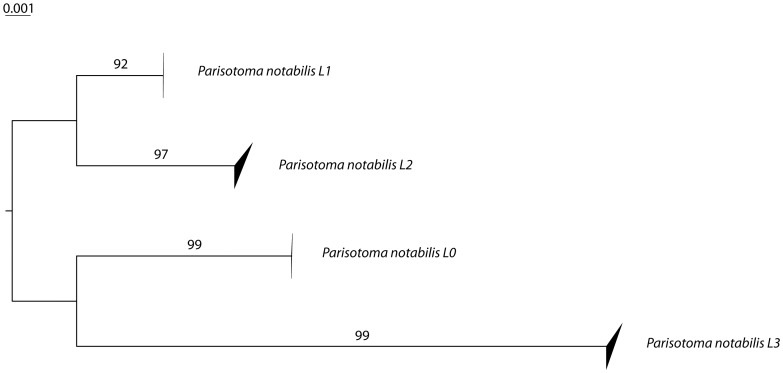
Neighbor-joining K2P distance tree for 28S (Bootstrap support values showed on the branches. Upper and lower side of the triangle represent the maximum and minimum of genetic distances within a species).

Four distinct COI lineages were detected within *P. notabilis*. Mean intraspecific and interspecific divergence among these 4 lineages were respectively 2.88% and 21.14% (Fig. 2 and 3, Table 2). Comparable distances were found among the other *Parisotoma* species included in the analysis with 0.54% for intraspecific variation and 26.4% for interspecific divergence (Table 2). The three populations more extensively sampled showed comparable intraspecific divergences with the lineages they belong to (*P. notabilis* L0 Hamburg 3.55%; *P. notabilis* L1 Guelph 2.92%; *P. notabilis* L2 Guelph 0.82%; *P. notabilis* L1 Paris 3.76%). Several lineages were found sympatric in some of the localities (Table 1). Only L0 was detected in the type locality in Hamburg.

**Table 2:** Intraspecific and interspecific K2P-pairwise distances (%).

#	Species	Intraspecific	Interspecific					
			1	2	3	4	5	6
1	*P. notabilis L0*	0.973						
2	*P. notabilis L1*	3.630	21.02					
3	*P. notabilis L2*	1.294	19.97	19.00				
4	*P. notabilis L3*	0.023	22.98	21.59	22.90			
5	*P. amurica*	0.000	21.65	21.05	21.17	21.68		
6	*P. ekmani*	2.111	25.30	23.88	26.17	24.48	26.10	
7	*P. hyonosenensis*	0.000	27.10	24.09	25.17	26.75	27.86	25.82

All the COI lineages of *P. notabilis* were retrieved with 28S. For the nuclear marker, the divergence between lineages was 2.24% and no intralineage variation was detected.

The neighbor joining trees produced for both genes show that the divergences among *P. notabilis* lineages are well supported (Fig. 2 and 3). The distribution of the four lineages in the various localities sampled is displayed in Table 1. All the individuals collected from the type locality clustered together in the lineage L0. Some of the lineages are sympatric in several localities (Table 1).

## Discussion

### Cryptic Diversity in *P. notabilis*

The four distinct lineages detected within the morphological boundaries of the species *P. notabilis* were supported by genetic divergences in both mitochondrial (COI) and nuclear markers (28S). The high divergences among *Parisotoma* species and *P. notabilis* lineages for COI matched the values observed between closely related [27], and even between more distant [39], [40] species of Collembola. The congruence with the nuclear gene 28S confirms the genetic individualization of these lineages, and suggests their specific status. On a morphological ground, such a result was unexpected, as this ubiquist species was so far considered as well-defined and homogeneous by modern taxonomists [10], [11], [12], [7], [2]. However, an unpublished dissertation [41] overlooked in recent literature, clearly showed that *P. notabilis* was constituted of different COI cryptic lineages. Several authors using various markers also established that different populations of the same species might be highly divergent genetically in Collembola [42], [43], [44], [45], [46], [47], [48], [49], [50], [51], [52]. In the present study, further elements of interpretation are gained by comparing high intraspecific divergences to levels of interspecific divergence in conspecific species. Recently, this method has also been used to detect cryptic lineages in six other species of Collembola [53]. Although morphological diversity was not re-evaluated among in most of these studies, some of them led to rehabilitate disused characters and disused names in species complexes: *Isotomurus palustris* [46], [48] and *Lepidocyrtus dispar/biphasis* [52]. This will not be possible in *P. notabilis*, as disused names are not based on disused characters.

**Figure 4:**
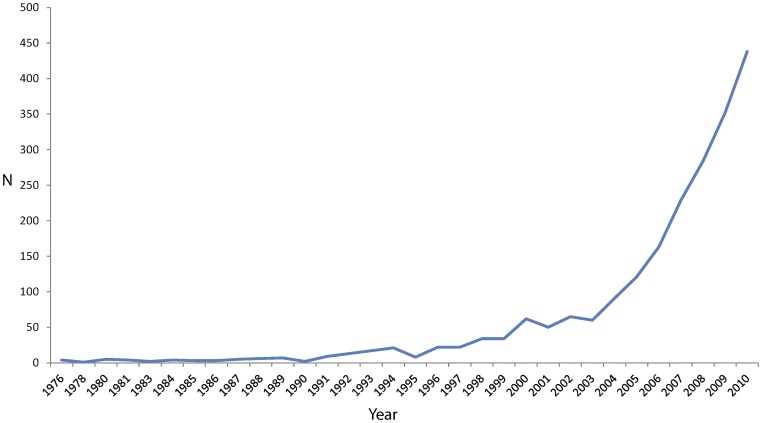
Bibliographic survey on cryptic diversity over the last 30 years (Source: Web of Knowledge).

### Retrieving the ‘True’ *P. notabilis*

One of the main problems raised by such results is to recognize which genetic entity should bear the species name. Here, the sequencing of specimens collected from the type locality (Hamburg, Germany), thus considered as representative of *P. notabilis* sensu stricto, allowed to resolve this issue. All these individuals clustered in the lineage L0. As a consequence, the species name *Parisotoma notabilis* should be assigned to this lineage. In this context, the critical importance of integrating DNA barcodes in new species descriptions through the sequencing of the holotype [54], [27] has to be emphasized. Indeed this could be much helpful in Collembola, where most widespread species are likely to be complexes of closely related forms [55]. Here, only one lineage was retrieved from the type locality, allowing to get easily to a conclusion. But this approach has limitations [53], as for instance, several lineages could occur in a species type locality, leaving as sole solution the sequencing of the holotype specimen, thus often bringing up ancient DNA issues because of the specimens age [54], [56].

### Naming the Other Species-level Lineages and Consequences for Previous Results

The second critical problem is to name the extra genetic entities recognized in this study. They are provisionally named after the lineage number (L1, L2 and L3) awaiting for a formal morphological description, although we were not, so far, able to distinguish them morphologically. Even if these lineages can be discriminated morphologically, the forms synonymized with *P. notabilis* (listed in [2]) will have to be checked, along with the DNA barcoding of populations from their type localities. This approach could possibly lead to the restoration of some of the synonyms as valid species names.

Meanwhile, the adoption of the provisory naming proposed here for lineages could allow their consistent use and characterization in various disciplines. This is critical as these lineages could potentially represent distinct species with different ecological and biological traits. Indeed, from the many examples in the literature showing discordance for pH preference [23], response to pesticides [15], [19], industrial pollution and heavy metal [20], [21], [15], [18], [22], the ecological homogeneity of the *P. notabilis* complex could have already been questioned. Actually, because of this overlooked situation, a considerable amount of information has been accumulated on *P. notabilis*, which turns out here to be a chimeric species composed of several distinct genetic entities. A similar situation has also been stressed in earthworms [57]. Awaiting for a better knowledge of the spatial and ecological distribution of the different lineages agglomerated in this complex, the previous results obtained so far for the nominal species *P. notabilis* should be considered with caution as they potentially bear on different lineages (see ‘*Geographical structuration’* section). In the worst-case scenario one single study could have involved several lineages at the same time as some of them were found to be sympatric in our dataset (Table 1). Further studies, identifing the different genetic lineages previously to experimentation, will help correcting this confusion by accumulating consistently information on the ecological and biological characteristics of these different genetic entities. In this respect, the provisional naming proposed here could facilitate such an initiative.

### Geographical Structuration

The distribution of *P. notabilis* lineages in Europe, beside some cases of sympatry, shows a clear geographical pattern of mostly parapatric distribution of lineages (Fig. 1). *Parisotoma notabilis* L0, the “true” *P. notabilis*, is restricted to Northern Europe; *P. notabilis* L1 is largely distributed in Southern Europe, and *P. notabilis* L2 mostly in the Alpine-Carpathic mountains range. This pattern suggests that the *P. notabilis* cited in regional studies of soil ecology may correspond to different lineages in different European regions. But such geographical structuration is not found in Northern America: in contrast with our European dataset, where sympatry is less frequent, the dataset at hand for this region shows that three different lineages are present in the same small and ecologically poorly diversified area of Eastern Canada (Table 1). Only one of the lineages, *P. notabilis* L3, has not been detected in North America so far, suggesting potential ecological or biological differences affecting its invasion capacity [58]. Further systematic sampling associated to DNA barcoding across the whole distribution area is needed to confirm and complete the observed geographical distributions, and to evaluate the spatial patterns of sympatry of the different lineages.

### Cryptic Diversity and Morphological Stasis

Due to the increasing use of molecular taxonomy tools, the number of publications on cryptic diversity has grown in the literature during the last ten years (Fig. 4). The number of cases reported in invertebrates experienced a fast increase [59], [60], [61], [62], [63], [64], [65], [66], [56], [67]. Often, either morphological examination initially failed to distinguish several good species within a nominal one, or the observed variability was considered as polymorphism. A broad survey revealed that cryptic diversity phenomenon is evenly distributed among major metazoan taxa and biogeographic regions when corrected for species richness and study intensity [68]. As most organisms surveyed do not dwell in extreme conditions, such a homogeneous distribution of the phenomenon marginalizes the hypothesis of strong environmental constraints driving morphological stasis [69] as a general explanation for cryptic diversity. In this respect, the *P. notabilis* complex uncovered here is a good example as none of its components dwell in highly constraining habitats.

An alternative hypothesis to explain cryptic diversity is that animals currently use chemical and auditory signals for sexual recognition, preventing morphological taxonomy based solely on visual observations to delineate accurately species [69]. But this does not apply here as *P. notabilis* is parthenogenetic with the exception of a Swedish population exhibiting rare male occurrence [70].

### Parthenogenetic Species

The parthenogeny of this species implies that the cryptic lineages found here are also potential parthenogenetic species. Our findings support this notion described in Bdelloid rotifers [71], [72] and Oribatid mites [73]. As predicted for such species [72], we found discrete clusters, reciprocally monophyletic for both nuclear and mitochondrial genes in *P. notabilis*. One of the main drivers of the asexual speciation is the diversifying selection due to niche adaptation [71]. The case of *P. notabilis* will have to be investigated in this respect, as the strong ecological discrepancies described in literature and the sympatric distributions of some of the lineages suggest that such a mechanism is likely responsible for the origin of this complex.

### Conclusion

This study established the evidence of multiple species-level lineages within one of the most ubiquist species of European Collembola. Most species of widespread European Collembola has been suspected to be complexes of closely related forms [56], and this has already been proved for some of them [54]. Our results support this assumption for *P. notabilis*, and advocate for a comprehensive molecular survey of the main species of Collembola currently used as models in various fields of investigations.
